# Supplementary material for: Dissolved Oxygen and Water Temperature Drive Vertical Spatiotemporal Variation of Phytoplankton Community: Evidence from the Largest Diversion Water Source Area
Source: Int J Environ Res Public Health. 2023 Feb 28;20(5):4307. doi: 10.3390/ijerph20054307 (PMC10002381; doi:10.3390/ijerph20054307)
Supplement: Supplementary file 1 [file ijerph-20-04307-s001.zip › ijerph-2214086-supplementary.pdf]

Supplemental Table S1. Location characteristics of sampling sites in the Danjiangkou Reservoir

Sample	Abbreviation	Maximum depth /(m)	Latitude	Longitude	Type
Heijizui	H	48	32°82′	111°53′	Danku entry site
Songgang	S	39	32°77′	111°66′	Wharf bay
Kuxin	K	48	32°74′	111°57′	Danku kuxin
Qushou	Q	32	32°65′	111°68′	Water diversion area of Middle Road project
Taizishan	T	48	32°66′	111°52′	Hubei and Henan border
Bashan	B	65	32°56′	111°48′	Hanku body
Langhekou	L	64	32°59′	111°31′	Han river entry site

Supplemental Table S2. Proportion of species in the Danjiangkou Reservoir

Phylum	Family	Genus	Species	Proportion
Chlorophyta	28	35	62	39.49%
Bacillariophyta	20	25	44	28.03%
Cyanobacteria	11	13	21	13.38%
Charophyta	1	2	3	1.91%
Miozoa	4	4	8	5.10%
Euglenozoa	2	3	7	4.46%
Cryptophyta	2	2	6	3.82%
Ochrophyta	3	3	5	3.18%
Haptophyta	1	1	1	0.64%
Total	72	88	157	100.00%

Supplemental Table S3. Test results of inter-subject effects of diversity indices in the Danjiangkou Reservoir

Index	Test value	Season	Layer	Season*layer
Total abundance	F	9.455	26.588	4.333
	p	<0.001	<0.001	<0.001
S	F	34.633	30.864	1.091
	p	<0.001	<0.001	0.361
H′	F	50.65	10.783	3.503
	p	<0.001	<0.001	<0.001

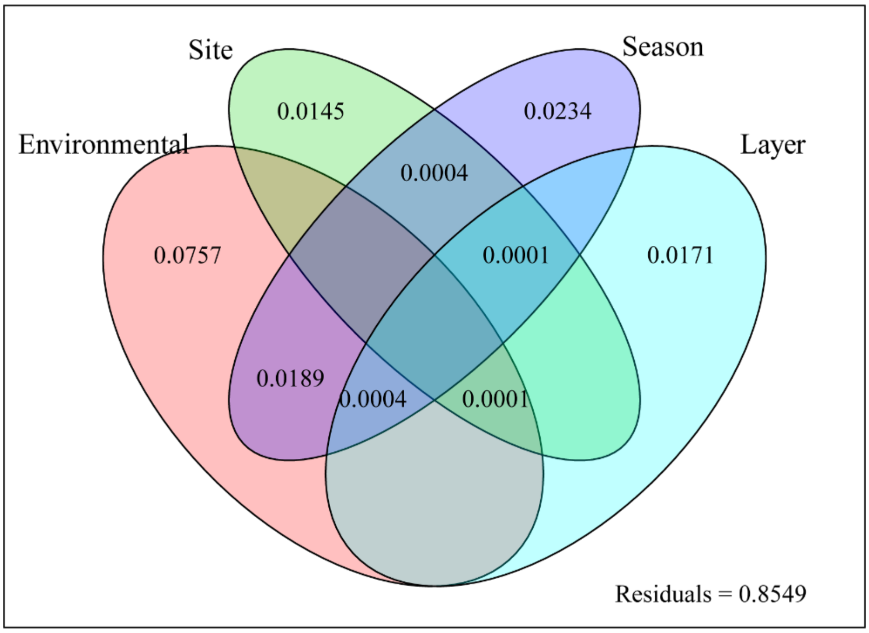

Supplemental Figure S1. Variation partitioning of phytoplankton community in the Danjiangkou Reservoir
